# Multi-organ spreading of *Actinobacillus pleuropneumoniae* serovar 7 in weaned pigs during the first week after experimental infection

**DOI:** 10.1186/s13567-018-0592-0

**Published:** 2018-09-25

**Authors:** Doris Hoeltig, Judith Rohde, Renate Frase, Florian Nietfeld, Karl-Heinz Waldmann, Peter Valentin-Weigand, Jochen Meens

**Affiliations:** 1 Clinic for Swine and Small Ruminants and forensic Medicine and Ambulatory Service, University of Veterinary Medicine, Foundation, Bischofsholer Damm 15, 30173 Hannover, Germany; 2 Institute for Microbiology, University of Veterinary Medicine, Foundation, Bischofsholer Damm 15, 30173 Hannover, Germany; 3 Innovative Veterinary Diagnostics (IVD GmbH), Albert-Einstein-Str. 5, 30926 Seelze, Germany; 4 Clinic for Swine, Department of Veterinary Medicine, Justus-Liebig-University Giessen, Frankfurter Str. 112, 35392 Giessen, Germany

## Abstract

*Actinobacillus* (*A*.) *pleuropneumoniae* is normally considered strictly adapted to the respiratory tract of swine. Despite this, scattered case reports of arthritis, osteomyelitis, hepatitis, meningitis or nephritis exist, in which *A. pleuropneumoniae* remained the only detectable pathogen. Therefore, the aim of this study was to investigate whether spreading to other organs than the lungs is incidental or may occur more frequently. For this, organ samples (blood, liver, spleen, kidney, tarsal and carpal joints, meninges, pleural and pericardial fluids) from weaners (*n* = 47) infected experimentally with *A. pleuropneumoniae* serovar 7 by aerosol infection (infection dose: 10.9 × 10^3^ cfu/animal) were examined by culture during the first week after infection. In addition, tissue samples of eight weaners were examined by histology and immunohistochemistry (IHC). *A. pleuropneumoniae* was isolated in all examined sample sites (86.7% pleural fluids, 73.3% pericardial fluids, 50.0% blood, 61.7% liver, 51.1% spleen, 55.3% kidney, 14.9% tarsal joints, 12.8% carpal joints, 27.7% meninges). These results were also obtained from animals with only mild clinical symptoms. IHC detection confirmed these findings in all locations except carpal joints. Histological examination revealed purulent hepatitis (*n* = 2), nephritis (*n* = 1) and beginning meningitis (*n* = 2). Isolation results were significantly correlated (*p* < 0.001) with the degree of lung colonization and, to a lower extent, with the severity of disease. Detection of *A. pleuropneumoniae* in peripheral tissues was significantly correlated to spleen colonization. In conclusion, multi-organ spreading of *A. pleuropneumoniae* serovar 7 strain AP 76 seems to occur more frequently during acute infection following effective lung colonization than previously thought.

## Introduction

*Actinobacillus* (*A.*) *pleuropneumoniae*, a Gram-negative bacterium belonging to the family *Pasteurellaceae*, is one of the most important respiratory tract pathogens in the pig industry. *A. pleuropneumoniae* is distributed worldwide and considered a primary pathogen that can cause severe respiratory disease. It affects the animal’s welfare due to peracute to chronic diseases, including severe fibrino-haemorrhagic and necrotizing pleuropneumonia with an increase in mortality [1, 2]. Therefore, it leads to severe economic losses in the pig industry which are due to the loss of animals, and costs of disease treatment as well as reduced performance during the chronic course of disease resulting in a prolonged fattening period of the animals [3, 4]. The severity of disease depends on several factors, including the infecting serovar, infection dose, co-infections, immune status and genetic background of the animal, as well as environmental factors [5–7]. In addition to its primary role in porcine pleuropneumonia *A. pleuropneumoniae* is also often involved in the development of the porcine respiratory disease complex, a co-infection of the respiratory tract of pigs [8–10]. In contrast to certain other porcine respiratory pathogens, e.g., *Haemophilus (H.) parasuis* [10, 11], *A. pleuropneumoniae* is considered a non-invasive lung pathogen [12] which solely affects lungs and pleura by spreading from the infected lung to the pleura via lymph vessels or oedematous fluid [13]. A bacteraemia is very rare [12, 13].

Despite its non-invasive character there are several case reports about non-respiratory clinical diseases in which *A. pleuropneumoniae* was the only detectable pathogen. These reports include cases of fibrino-purulent arthritis and necrotizing osteomyelitis [14], granulomatous hepatitis [15], meningitis and, nephritis [16] as well as endocarditis and fibrinous peritonitis [17]. These recurring case reports prompted us to find out if *A. pleuropneumoniae* can be detected more frequently within different body tissues during the acute phase of disease. For this, we investigated possible spreading of *A. pleuropneumoniae* to body tissues other than the lungs in experimentally infected weaners.

## Materials and methods

### Animals and housing conditions

To investigate the possible spreading of *A. pleuropneumoniae* to other organs than lungs forty-seven weaners experimentally infected with *A. pleuropneumoniae* serovar 7 from another infection trial were chosen by simple randomization. This infection trial was originally conducted in the context of evaluation of genetically determined susceptibility of pigs towards the pathogen. The pigs were kept in accordance with the Guidelines for Protection of Vertebrate Animals used for experimental and other Scientific Purposes, European Treaty Series, nos. 123/170. Study setup and housing of the animals were approved by an independent local committee on ethics. The pigs were fed a commercial standardized diet and were provided 2.5 kg of hay flakes per group (^®^AGROBS Pre Alpin Wiesenflakes, Co. AGROBS, Degerndorf, Germany) per day as material for rooting and manipulation. The group size was 8 to 10 pigs on 8 m^2^. Ambient temperature was 27.5 °C ± 1.9 °C (MEAN ± SD) and humidity was 33.2% ± 12.5% (MEAN ± SD). Within each pen 2.8 m^2^ were covered with a rubber mat, heated by two infrared lamps, for bedding area. Allocation to the housing groups was also performed by simple randomization. Drinking quality water was constantly available. The pigs arrived 3 weeks prior to infection for an adequate adaptation to diet and new environment.

### Experimental infection

Prior to experimental infection all pigs underwent a general clinical examination and bronchoalveolar lavage fluids (100 mL 0.9% NaCl-solution ad us. vet., Co. WDT, Garbsen, Germany) as well as serum samples were taken. The bronchoalveolar lavage fluid was examined by bacteriological culture and PCR [18] for the absence of *A. pleuropneumoniae*. Bronchoalveolar lavage was performed under general anesthesia with 20 mg/kg Ketamine i.m. (Ketamin 100 mg/mL^®^, Co. CP-Pharma, Burgdorf, Germany) and 2 mg/kg Azaperon i.m. (Stresnil^®^, Co. Janssen-Cilag GmbH, Baar, Switzerland). Serum samples were analyzed by ApxIV-ELISA (IDEXX APP-ApxIV Ab Test^®^, Co. IDEXX Laboratories, Maine, USA). Only pigs confirmed as clinically healthy and negative for *A. pleuropneumoniae* by direct and indirect screening procedures were included for infection. At the time of infection the pigs were 7 weeks of age and had a mean bodyweight of 12.6 kg ± 2.1 kg.

The experimental infection was done by aerosol infection with a total exposure time of 30 min [6]. For this, approximately 1 × 10^5^ bacteria of *A. pleuropneumoniae* AP76 serovar 7 were nebulized resulting in an aerosol concentration of 1 × 10^2^ colony forming units (cfu) per liter aerosol. Based upon a mean tidal volume of pigs of 9 mL/kg BW [19] the mean infection dose inhaled per pig was 10.9 × 10^3^ cfu. The group size for infection was five to six pigs. After infection the pigs were clinically monitored every 2 h within the first 48 h after infection. Subsequently, the clinical examination was done twice a day. Exit criteria for euthanasia of the pigs were defined to minimize the suffering of the infected animals. Exit criteria were as follows: a respiratory score (Table 1) of 3; a depression score (Table 1) of 3; a rectal body temperature > 42.0 °C; a respiratory or depression score greater than 1 and a body temperature < 37.5 °C; a body temperature > 40.3 °C and/or a depression score and respiratory score > 1 for more than two consecutive days; a body temperature > 40.3 °C and/or depression score and respiratory score > 0 for more than two consecutive days from day three post infection onwards; any unpredictable event, reaction to treatment or disease leading to a moderate reduction of general condition or inducing pain for more than 48 h; any unpredictable event, reaction to treatment or disease leading to severe reduction of general condition or inducing pain.

**Table 1:** **Scoring systems used for the application of exit criteria**

Scoring systems	Scoring scale based upon clinical symptoms			
	0	1	2	3
Respiratory score	No clinical signs of respiratory disease	Breathing frequency of 35–55/min and/or occasional coughing	Breathing frequency of 56–90/min and/or multiple coughing periods within 10 min and dyspnea	Breathing frequency > 90/min and cyanosis or gasping or open-mouth breathing or breathing frequency > 90/min and cyanosis and gasping or open-mouth breathing
Depression score	Active, alert, normal ingestion	Calm, alert, decreased ingestion	Dull, increased recumbence, increased reaction time, still moving to the feeding trough but without or only small feed intake or dull, sitting like a dog, increased reaction time, still moving to the feeding trough but without or only small feed intake	Apathetic, no reaction to stimulation and/or shaky movements without lying down and/or standing with head down without lying down and/or vomiting and/or foam around nostrils and mouth

### Clinical investigation

The monitoring of the clinical signs consisted of an assessment of posture, behavior, feed intake, rectal body temperature, vomiting, breathing noise, dyspnea, respiratory frequency, coughing and skin color. The results of the clinical examinations were transferred to an objective clinical scoring system [20]. According to this scoring system the pigs were assessed as non-diseased when showing scoring points ≤ 1.3; as mildly diseased when showing scoring points > 1.3 ≤ 12.5; as moderately diseased with scoring points > 12.5 ≤ 23.7 and as severely diseased when reaching scoring points > 23.7 on day seven post-infection (pi).

### Necropsy and bacteriological examination

Seven days post infection (or earlier in case of withdrawal due to exit criteria) the pigs were euthanized by intravenous application of 80 mg/kg pentobarbital (Euthadorm^®^, Co. CP Pharma GmbH, Burgdorf, Germany). Necropsy was performed and the degree of lung lesions was assessed using a lung lesion score [21]. For this score a schematic map of the lung is divided into triangles. According to the assessed lung lesions triangles are marked. Each lung lobe can reach a maximum score of 5 resulting in an overall maximum score of 35. The degree of lung lesions was classified as mild with scoring points > 0 ≤ 11.6; as moderate with scoring points > 11.6 ≤ 23.2 and as severe when reaching scoring points > 23.2.

For the isolation of *A. pleuropneumoniae* tissue samples were taken from 13 different locations. In total seven lung tissue samples of defined positions located in the outer third of each of the seven lung lobes, three central organ samples including liver, spleen and left kidney and three peripheral tissue samples including swabs from the meninges as well as the carpal and tarsal joints of the animals were collected. All tissue samples had a size of approximately 1 cm^2^. In cases where there were no macroscopic lesions in the defined locations of the lung lobes but in other parts of the lung lobes additional samples from the macroscopically altered regions were taken. Tissue samples and swab samples were plated on *A. pleuropneumoniae*-selective blood agar [22] using the quadrant streaking method. Abundance of growth was assessed semi-quantitatively. Additionally from 15 pigs pleural and pericardial exudates were sampled and evaluated regarding volume and number of viable *A. pleuropneumoniae* cells. The second was determined by plating tenfold serial dilution on *A. pleuropneumoniae*-selective blood agar. Bacterial isolates were identified as *A. pleuropneumoniae* by PCR amplification of the apxIV gene [23].

The amount of growth for each organ or swab sample was transferred to a scoring system (0 = no growth; 1 = sparse growth; 2 = moderate growth; 3 = heavy growth). For the lung tissue samples the level of isolation from the lungs was translated into a combined isolation score [24]. For this purpose the amount of growth from all lung tissue samples was added and divided by the total number of lung tissue samples. Results were classified as low-grade isolation level (score 0–1), moderate (> 1 and ≤ 2) and high-grade (> 2–3).

Additionally blood samples were taken from the Vena jugularis at the time of euthanasia from 26 pigs. These blood samples were inoculated into SIGNAL blood culture systems (SIGNAL blood culture system^®^; Co. Oxoid, Basingstroke, Hampshire, UK) and bottles with medium in the growth indicator device were subcultured for *A. pleuropneumoniae*. From 23 of these pigs 0.5 mL of the taken blood sample was directly plated on *A. pleuropneumoniae*-selective blood agar [22] using the quadrant streaking method, too. For these directly plated blood samples the abundance of growth was assessed semi-quantitatively like for the other tissue samples.

### Histological examination and immunohistochemistry

A total of eight pigs were chosen randomly for additional histological examination and immunohistochemical (IHC) investigation. Tissues collected at necropsy for these investigations included liver, spleen, pericardium, peritoneum, kidney, cerebrum, and carpal and tarsal synovialis. Tissues were fixed by immersion in 10% neutral buffered formalin for 1–7 days and routinely processed in an automated tissue processor, embedded in paraffin, sectioned at 3 µm, and stained with hematoxylin and eosin. Additional sections were mounted on poly-l-lysine-coated slides (SuperFrost Plus^®^; Thermo Fischer Scientific, Dreieich, Germany) for immunohistochemical evaluation.

A biotin-free horse-radish-peroxidase (HRP)-polymer-based detection system (Ultravison LP HRP, Thermo Fisher Scientific, Dreieich, Germany) was used for immunohistochemical detection of *A. pleuropneumoniae* antigen. Antigen retrieval was achieved by immersion of the deparaffinized rehydrated slides in preheated EDTA buffer (pH 8) for 15 min at 90 °C in a steamer. After cooling down to at least 60 °C the slides were washed 3 times in Tris buffer containing 0.05% Tween 20 (Carl Roth GmbH, Karlsruhe, Germany) at room temperature. Then they were blocked for 7 min at room temperature with Ultra V blocking solution (Thermo Fisher Scientific, Dreieich, Germany) and incubated with a mixture of two primary antibodies. These primary antibodies had been produced previously in rabbit using recombinant Outer Membrane Lipoprotein A (OmlA) of *A*. *pleuropneumoniae* serovar 1 and 5 isolates [25, 26]. The ability of the anti-OmlA serovar 1 serum to detect *A. pleuropneumoniae* serovars 1–12 and 14 and the ability of the anti-OmlA serovar 5 serum to detect *A. pleuropneumoniae* serovar 5–8 and 10 had been verified previously during western blot validation assays performed at the IVD laboratory using isolates of *A. pleuropneumoniae* serovars 1–15. No unspecific cross-reaction of the primary antibodies was detected in western blot and dot blot assays with *Actinobacillus porcitonsillarum*, *A. minor*, *A. porcinus*, *A. indolicus*, *A. suis*, *Haemophilus parasuis* or *Pasteurella multocida.* After primary antibody incubation overnight at 4 °C the slides were rinsed 3 times in Tris buffer containing 0.05% Tween 20 (TBS-T) and incubated with the secondary antibody (Primary Antibody Enhancer of the Ultravision Detection LP kit mentioned above) for 15 min at room temperature. Following 3 washing steps in TBS-T the slides were incubated with the HRP-polymer for 30 min at room temperature. After 3 washing steps in TBS-T the slides were immersed in 3% hydrogen peroxide for 10 min in order to block endogenous peroxidase. Followed by 3 washing steps in TBS-T the slides were treated with 3,3-diaminobenzidine tetrahydrochloride (DAB) chromogen plus substrate solution (Thermo Fisher Scientific, Dreieich, Germany) for 10 min, washed again in TBS-T and counterstained with hematoxylin Gill 2.

### Statistical methods

The collected data were transferred to an Excel^®^ based database (Co. Microsoft Cooperation, Dublin, Ireland). Statistical analyses were carried out using Excel^®^ and IBM SPSS Statistics^®^ (Co. IBM Deutschland GmbH, Ehningen, Germany). For correlation analysis Spearman Rank Correlations were calculated. Correlations of < 0.05 were classified as significant and of < 0.01 as highly significant.

## Results

Based on the clinical scoring, 7 of 47 infected pigs were classified as non-diseased, 21 pigs as mildly diseased, 3 pigs as moderately diseased and 16 pigs as severely diseased. Nineteen pigs had to be euthanized due to acute exit criteria and 1 pig due to chronic exit criteria. Twenty-seven pigs were euthanized on day 7 pi. The degree of gross lung lesions was mild in 13 pigs, moderate in 12 pigs and severe in 19 pigs. In 3 pigs there were no macroscopic lung lesions. For the lung tissue, isolation score of *A. pleuropneumoniae* was low-grade in 9 pigs, moderate in 13 pigs and high-grade in 22 pigs. *A. pleuropneumoniae* could not be isolated from the lungs of 3 pigs.

An overview of the isolation results from other tissues than the lungs is shown in Table 2. *A. pleuropneumoniae* could be cultured from 50% of the blood samples by blood culture, from 43.5% of the blood samples by direct plating, from 61.7% of the liver samples, 51.1% of the spleen samples, 55.3% of the kidney samples, 14.9% of the tarsal joints swabs, 12.8% of the carpal joints swabs and 27.7% of the meningeal swabs. The pleural fluid was positive for *A. pleuropneumoniae* in 86.7% and the pericardial fluid in 73.3% (Table 3). The bacterium could not be re-isolated from any of these locations in pigs classified as non-diseased. Within the sampled pleural fluids the volumes ranged from 1 to 230 mL and the isolation results of these samples ranged from 9.5 × 10^3^ to 1.3 × 10^9^ cfu. The volumes of the pericardial fluids ranged from 1 to 70 mL and here the isolation results ranged from 2 to 2.0 × 10^8^ cfu. From the tissue samples isolation scores for *A. pleuropneumoniae* ranged from low-grade to high-grade whereas the isolation score was only low-grade (76.9%) and moderate (23.1%) from meningeal swabs and only low-grade (100%) from tarsal and carpal joint swabs. *A. pleuropneumoniae* could be cultured from all these locations not only from pigs classified as moderate or severely diseased but also from pigs classified as mildly diseased (blood: 2 pigs; liver: 15 pigs; spleen: 14 pigs; kidney: 13 pigs; tarsal swabs: 7 pigs; carpal swabs: 6 pigs; meningeal swabs: 10 pigs). Isolation was possible from animals euthanized due to exit criteria (blood: 11 pigs; liver: 16 pigs; spleen: 16 pigs; kidney: 14 pigs; tarsal joints: 5 pigs; carpal joints: 5 pigs; meninges: 9 pigs) as well as from animals euthanized 7 days pi (blood: 2 pigs; liver: 13 pigs; spleen: 8 pigs; kidney: 12 pigs; tarsal joints: 2 pigs; carpal joints: 1 pig; meninges: 4 pigs). *A. pleuropneumoniae* was not detected in organ samples of pigs that showed no colonisation of the lungs. It was mainly detected in tissues other than the lungs if the animals had a moderate or high-grade score of isolation from lung tissue, too. There was no detection of *A. pleuropneumoniae* in blood, kidney, tarsal, carpal and meningeal samples from animals with low-grade isolation from lung tissue. For liver and spleen samples only one pig with a low-grade lung isolation result was culturally positive.

**Table 2:** **Isolation of** ***A. pleuropneumoniae*** **from central and peripheral organ tissues and corresponding severity of disease during acute infection**

	Sample	Samples with positive isolation results (%)	Degree of re-isolation (number of animals)			Number of animals with isolation of *A. pleuropneumoniae* in the respective organs								
						Degree of clinical disease			Degree of lung lesions			Degree of isolation from the lungs		
			Low-grade	Moderate	High-grade	Mild	Moderate	Severe	Mild	Moderate	Severe	Low-grade	Moderate	High-grade
	Blood; blood culture (*n* = 26)	50.0 (*n* = 13)	–	–	–	4	3	6	2	2	9	0	2	11
	Blood; direct plating (*n* = 23)	43.5% (*n* = 10)	2	5	3	3	3	4	2	2	6	0	2	8
Central organ samples	Liver (*n* = 47)	61.7 (*n* = 29)	15	8	6	14	3	12	5	10	14	1	8	20
	Spleen (*n* = 47)	51.1 (*n* = 24)	14	5	5	9	3	12	2	8	14	1	6	17
	Kidney (*n* = 47)	55.3 (*n* = 26)	13	8	5	12	3	11	4	10	12	0	7	19
Peripheral organ samples	Tarsal joints (*n* = 47)	14.9 (*n* = 7)	7	0	0	2	1	4	0	3	4	0	2	5
	Carpal joints (*n* = 47)	12.8 (*n* = 6)	6	0	0	1	1	4	0	2	4	0	1	5
	Meninges (*n* = 47)	27.7 (13)	10	3	0	4	1	8	2	2	9	0	4	9

**Table 3:** **Detection of** ***A. pleuropneumoniae*** **in pleural and pericardial fluids and corresponding severity of disease during acute infection**

	Degree of clinical disease				Degree of lung lesions			
	Non-diseased	Mild	Moderate	Severe	No lesions	Mild	Moderate	Severe
Number of animals (*n* = 15)	1	8	1	5	0	3	5	7
*Pleural fluid*								
Min. volume (mL)	1	1	30	15	–	1	1	15
Max. volume (mL)	1	35	30	230	–	13	35	230
Mean volume ± SD (mL)	–	11.3 ± 11.4	–	87.0 ± 87.7	–	7.0 ± 6.4	13.8 ± 13.7	69.3 ± 77.8
Min. cell count (cfu/mL)	0	0	4.2 × 10^7^	9.7 × 10^7^	–	0	0	4.2 × 10^7^
Max. cell count (cfu/mL)	0	2.7 × 10^8^	4.2 × 10^7^	1.3 × 10^9^	–	1.5 × 10^7^	2.7 × 10^8^	1.3 × 10^9^
Mean cell count ± SD (cfu/mL)	–	5.9 × 10^7^ ± 9.7 × 10^7^	–	4.1 × 10^8^ ± 5.0 × 10^8^	–	7.5 × 10^6^ ± 8.6 × 10^6^	6.4 × 10^7^ ± 1.2 × 10^8^	3.2 × 10^8^ ± 4.4 × 10^8^
*Pericardial fluid*								
Min. volume (mL)	5	1	4	4	–	3	1	4
Max. volume (mL)	5	65	4	70	–	18	65	70
Mean volume ± SD (mL)	–	13.5 ± 21.6	–	29.0 ± 27.5	–	10.5 ± 8.1	15.6 ± 27.8	22.6 ± 25.0
Min. cell count (cfu/mL)	0	0	1.2 × 10^6^	8.7 × 10^4^	–	0	0	8.7 × 10^4^
Max. cell count (cfu/mL)	0	4.0 × 10^5^	1.2 × 10^6^	2.0 × 10^8^	–	17	1.5 × 10^2^	2.0 × 10^8^
Mean cell count ± SD (cfu/mL)	–	5.0 × 10^4^ ± 1.4 × 10^5^	–	4.0 × 10^7^ ± 8.9 × 10^7^	–	8.5 ± 9.8	32.4 ± 65.9	2.9 × 10^7^ ± 7.5 × 10^7^

**Figure 1:**
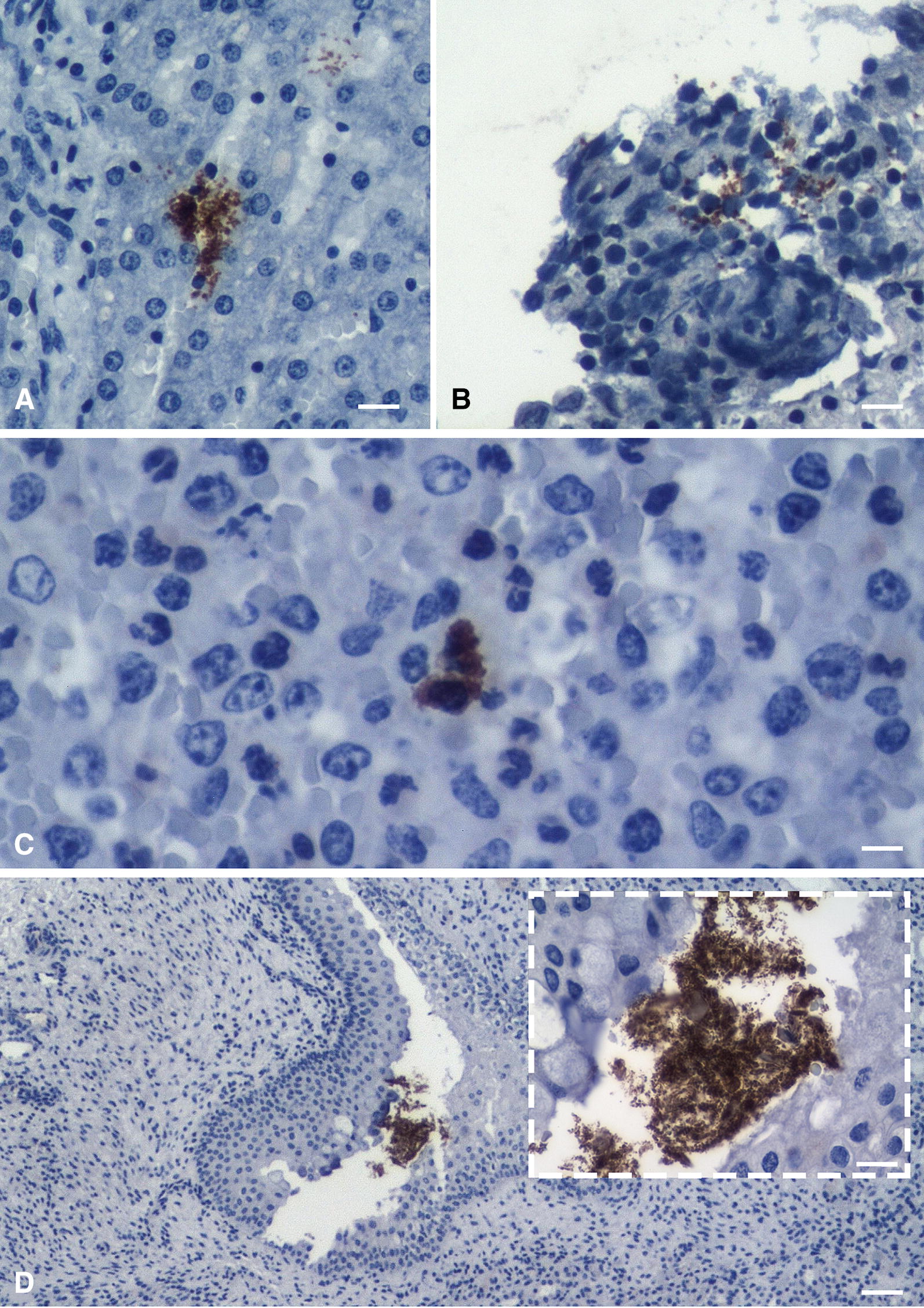
**Detection of** ***A. pleuropneumoniae*** **by immunostaining.** Detection in different body tissues during acute infection using a Horseradish-Peroxidase-(HRP)-polymer based detection system with DAB chromogen and Hematoxylin Gill 2 counterstain. **A** Liver (20-fold magnification): Note oligofocal detection of coccoid bacteria (brown) with *A. pleuropneumoniae* antigen within sinuisoids. **B** Pericard (20-fold magnification): Note detection of coccoid bacteria (brown) with *A. pleuropneumoniae* antigen within superficial exudate containing karyolytic leukocytes some of which are elongated (so called *oat cells*) which is characteristic of *A. pleuropneumoniae*. **C** Spleen (40-fold magnification): Note neutrophilia of the red pulp and detection of *A. pleuropneumoniae* antigen (brown) within two macrophages. **D** Kidney (4-fold magnification): Note colony of small rod-shaped bacteria with *A. pleuropneumoniae* antigen (brown) within the pelvis. Inlet: higher (40-fold) magnification of the bacterial colony.

**Figure 2:**
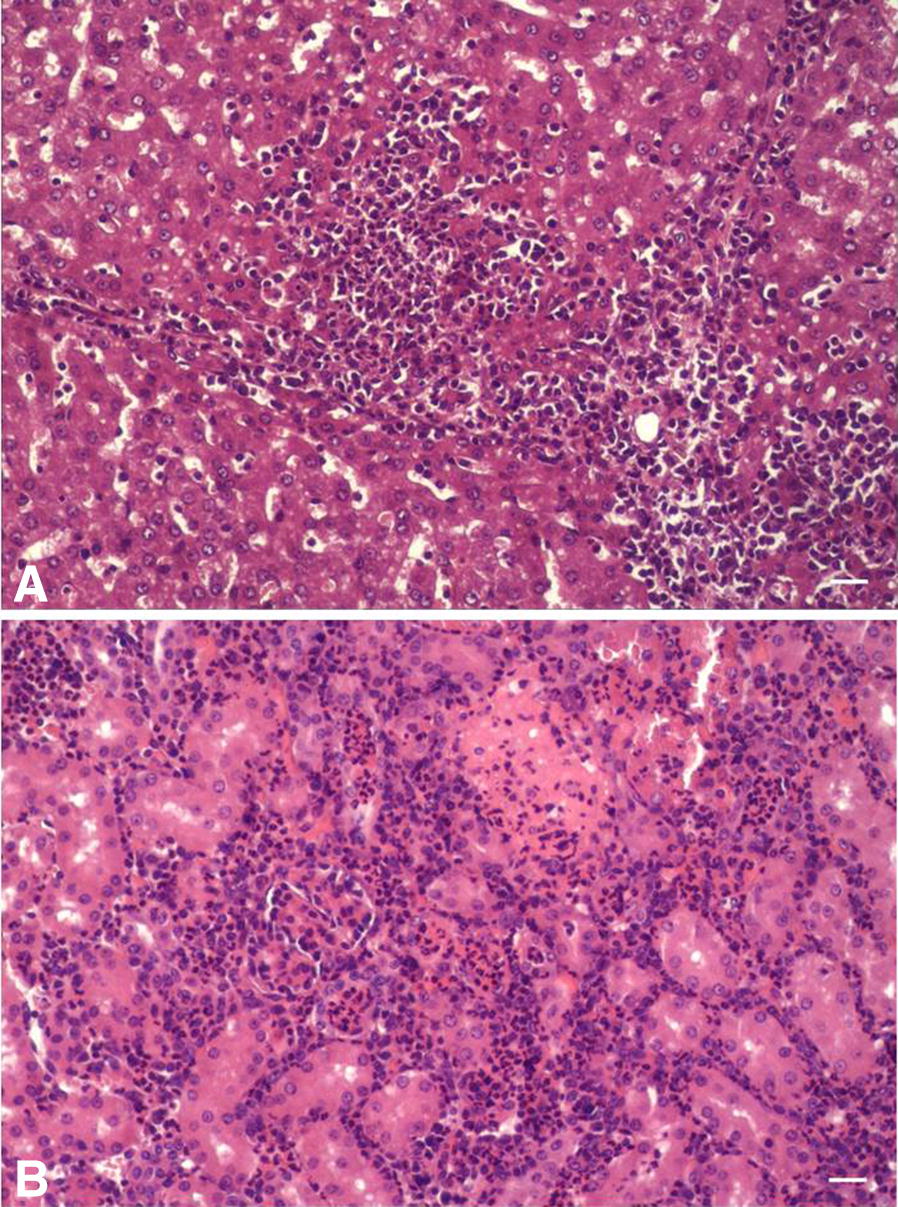
**Histological lesions in liver and kidney tissue after experimental infection with** ***A. pleuropneumoniae.*** 10-fold magnification, hematoxylin–eosin staining. **A** Liver: multifocal, acute, mild to moderate purulent hepatitis **B** Kidney: focal, acute mild to moderate embolic fibrino-purulent nephritis.

From the eight animals chosen for histological and IHC examination three were euthanized due to exit criteria and five were euthanized at day 7 pi. Two were clinically classified as non-diseased, four as mildly diseased and two as severely diseased. By IHC examination *A. pleuropneumoniae* was detected in all eight spleen samples (oligofocal within the red splenic pulp), five liver samples (focal to oligofocal, mainly sinusoidal and intravascular), three kidney samples (focal, intertubular, within the renal pelvis and intravascular embolism), two tarsal samples (focal as intravascular embolus), two meningeal samples (focal, leptomeningeal and pachymeningeal) and in none of the carpal samples. *A. pleuropneumoniae* was detected on the inner surface of the parietal pericardium in five animals (Figure 1). These positive results were obtained from animals euthanized due to exit criteria (liver: 3 pigs; spleen: 3 pigs; tarsal joints: 1 pig; meninges: 2 pigs) as well as from pigs euthanized at day 7 pi (liver: 2 pigs; spleen: 5 pigs; tarsal joint: 1 pig; meninges: 6 pigs). In the kidney samples *A. pleuropneumoniae* was only detected on day 7 pi by IHC. Although the macroscopic examination revealed no gross lesions in other organs than the lung the histological examination revealed a hypertrophy of the Schweigger-Seidel-sheaths and an acute diffuse moderate to severe hyperaemia in all spleen samples, a moderate neutrophilia in five spleen samples and a hypertrophy of the reticulocytes as well as an increased number of apoptotic cells in two spleen samples. Two pigs had a multifocal, acute and mild to moderate purulent hepatitis (Figure 2), one pig showed a focal, acute mild to moderate embolic fibrinous purulent nephritis (Figure 2) and two pigs showed a focal accumulation of neutrophilic granulocytes within Dura mater and cerebrum.

The histological examination of all seven lung samples revealed a severe acute fibrino-haemorrhagic and necrotizing pleuropneumonia for the two pigs that had been clinically classified as severely diseased when chosen for IHC. For two pigs with a mild clinical manifestation nonetheless a severe acute fibrino-haemorrhagic and necrotizing pleuropneumonia was detected but only in the lung tissue sample from the right caudal lung lobe. The third pig with a mild clinical disease showed mild to moderate fibrino-haemorrhagic and necrotizing lung lesions. In lung samples showing necro-haemorrhagic lesions, coccoid bacteria were detected in particular closely related to and within the lymph vessels. The fourth pig with a mild clinical disease and one of the two pigs that had been classified as clinically healthy showed no histological lesions associated with *A. pleuropneumoniae* infection. The second pig that was clinically classified as non-diseased showed a focal acute mild fibrinous pleuritis in the Lobus accessorius including pleural exudate composed of neutrophils, macrophages, fibrin and erythrocytes.

**Figure 3:**
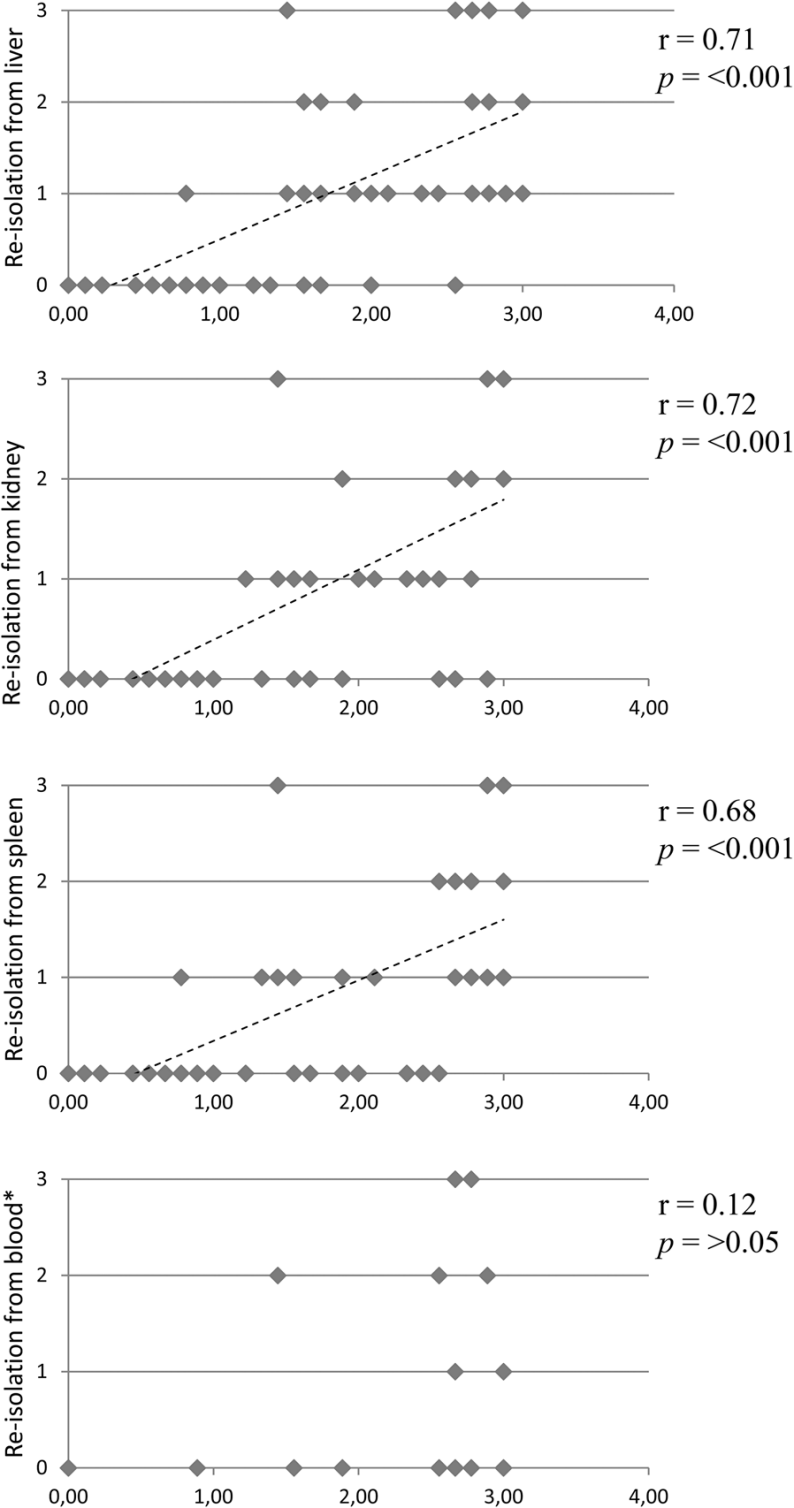
**Correlation analysis of isolation from lung tissue and central organ tissues.** X-axis: Score quantifying the degree of isolation from seven lung tissue localizations with 0 = no isolation, > 0–≤ 1 = low-grade isolation, > 1–≤ 2 = moderate isolation, > 2 = high-grade isolation. Degree of isolation from different central organ tissue is displayed on the y-axis as isolation score with 0 = no isolation, 1 = low-grade isolation, 2 = moderate isolation, 3 = high-grade isolation; diagonal lines: balance lines for degree of correlation; r: concordance value, *p*: significance value; concordance and significance calculated by Spearman Rank analysis; *results were obtained by direct plating of blood samples on *A. pleuropneumoniae*-selective blood agar [21] using the quadrant streaking method.

There was no significant correlation, either with the degree of clinical disease or with the degree of isolation from the lungs, the lung lesion score or the detection in other organ samples (Figure 3) for the detection of *A. pleuropneumoniae* in blood by either method (blood culture or direct plantings).

Volumes of the effusions and *A. pleuropneumoniae* cfu within the pleural fluids were highly significantly correlated with the degree of clinical disease and the degree of lung lesions (volume: *p*^disease^ = 0.005, *p*^lesions^ ≤ 0.001; cell count: *p*^disease^ = 0.004, *p*^lesions^ = 0.001). Regarding the pericardial fluids there was no significant correlation between fluid volumes and the degree of clinical disease or lung lesions developed during acute infection (*p*^disease^ = 0.264; *p*^lesions^ = 0.129). However, there was a highly significant correlations between both, the degree of clinical disease and the degree of lung lesions, and the amount of viable *A. pleuropneumoniae* cells within the pericardial fluids (*p*^disease/lesions^ = 0.001).

The detection of *A. pleuropneumoniae* by bacteriological culture within the inner organ samples (liver, spleen, kidney) was highly significantly correlated with the degree of isolation from the lung tissue (*p* ≤ 0.001; Figure 3). The detection in liver and spleen was also highly significantly correlated with the degree of clinical disease (spleen: *p* ≤ 0.001; liver: *p* = 0.003). The same was found for the isolation from spleen tissue and the lung lesion score (*p* = 0.001). For the detection in the kidney and the clinical score (*p* = 0.013) as well as for the lung lesion score and the isolation from liver (*p* = 0.016) and kidney (*p* = 0.049), respectively, there was a significant correlation.

For the peripheral swab samples (carpal joints, tarsal joints and meninges) there was a highly significant correlation between the detection of the bacterium in the meninges and the degree of clinical disease (*p* = 0.004). There was also a significant correlation between the isolation from the lungs and the isolation from tarsal joints (*p* = 0.032) and meninges (*p* = 0.019) as well as for the detection in the meninges and the lung lesion score (*p* = 0.039).

Regarding the isolation of *A. pleuropneumoniae* from inner organ tissues and peripheral organ swab samples, the isolation was highly significantly correlated between spleen and all peripheral samples (carpal joints: *p* = 0.010; tarsal joints: *p* = 0.004; meninges: *p* = 0.004) as well as between tarsal joints and kidney (*p* = 0.009), respectively. There was a significant correlation between the detection in carpal joint swabs and liver (*p* = 0.040) or kidney (*p* = 0.018) as well as between tarsal joint swabs and liver (*p* = 0.024) and between liver and meninges (*p* = 0.047).

## Discussion

The detection of *A. pleuropneumoniae* in pleural und pericardial fluids of pigs during acute infection are in accordance with earlier studies on porcine pleuropneumonia pathogenesis describing the mechanism of spreading of the pathogen within the pleural cavity not only via lymph vessels but also by oedematous fluids [13] and also describing the incidence of viable bacteria in pericardial fluids in cases of severe pleuropneumonia [27]. Though there was a significant correlation between the degree of clinical disease and lung lesions and detected cfu within the pericardial fluids, we could show, that, in contrast to Nicolet and König [27], *A. pleuropneumoniae* does not only appear in the pericardial fluids of animals developing a severe disease after infection, but also in the pericardial fluids of pigs only showing mild clinical symptoms and mild lung lesions. Since *A. pleuropneumoniae* strain AP 76 could be isolated from all tested organ locations, it can be concluded that this strain spreads regularly within the whole body of the pig during the acute phase of infection. Notably, an examination by PCR might have been more sensitive [28] and, thus, might have even revealed higher detection rates in other tissues than the lungs, the isolation by bacteriological culture in the present study demonstrates the viability of the detected *A. pleuropneumoniae* isolates and avoids that the detection might be based on fragments processed by the immune system [29]. As *A. pleuropneumoniae* antigen was also detected by IHC intravascular, intralesional, and intracellular within macrophages in different tissue samples, contamination of the samples during the sampling procedure can be excluded. Given that *A. pleuropneumoniae* was detected within the blood samples both by blood culture and direct plating as well as in intravascular emboli in the IHC examination it can be assumed that the spreading within the organism was due to bacteraemia. Since *A. pleuropneumoniae* is normally considered to be non-invasive [12] the bacterium might reach the bloodstream due to a damage of the blood vessel endothelium within areas of necrotizing pneumonia. However, in the lung samples of three of the animals examined by IHC and histology no damage of the blood vessel endothelium could be detected although the bacterium was detected within other organ tissues. The fact that there was no positive correlation between the detection in blood and the detection in any other organ tissues might, therefore, also be considered as a hint that the bloodstream might not be the first or only way for spreading. Nevertheless, it should be taken into account that bacteraemia is not always detected by blood culture and that a minimum of three samples per patient are needed to reach a sensitivity of 96% [30]. The histological detection of the bacteria notably adjacent and inside of the pulmonary lymph vessels suggests that spreading takes place mainly via the lymph system. In this case the pathogen might reach the blood stream subsequently via the thoracic duct at the venous angle. In conclusion, it remains to be clarified whether the spreading of *A. pleuropneumoniae* mainly occurs via the lymph system, as stated for the spreading from the lungs to the pleura [13] or mainly by the blood system. Direct passage to the blood stream might also be an incidental event within damaged lung tissue. Nevertheless, the results suggest that pathogenic isolate AP76, in contrast to previous reports on the pathogenesis of *A. pleuropneumoniae* infection, seems to have an invasive capacity. The histological findings indicate that in most cases during acute infection *A. pleuropneumoniae* seems to be harboured within different organs without causing any lesions or immune reactions. However, this investigation also reveals mild cases of nephritis and hepatitis, as well as findings of indicative possible beginnings of meningitis. These results are in accordance with the published case reports in which *A. pleuropneumoniae* was identified as the causative agent of clinical relevant hepatitis [15], meningitis and nephritis [16]. The detection in the tarsal and carpal swabs also might indicate an emigration to the joints even if a minimal contamination of the synovia with blood during sampling cannot be excluded with certainty. However, the fact that the agent was detected in these locations corresponds to a reported case of fibrino-purulent arthritis and necrotizing osteomyelitis [14], although, no alterations could be detected by histological examination of the joint capsules in our samples.

So far, we know hardly anything about virulence mechanisms involved in causing tissue alterations in organs other than lungs or, how long the bacterium survives in these organs and how it escapes the immune system especially during bacteraemia. Combining the IHC results and the results of the correlative analysis it appears that the degree of the colonisation of the lungs, as well, as the colonisation of the spleen seems to play a central role for the spreading of strain AP76 within the pig’s organs.

Regarding the results presented here, regularly multi-organ spreading within the pig seems to be proven for at least *A. pleuropneumoniae* serovar 7 strain AP67 used in this experiment. Whether this applies to other isolates and other serovars, too, still needs to be explored. However, the fact, that the strains identified in the published case report on other organ diseases include strains of serovar 2 [14, 15, 17], serovar 6 [16, 17] and serovar 3 [31] suggests that the ability for spreading within the whole organism of the pig might be held by several *A. pleuropneumoniae* strains.

*Actinobacillus pleuropneumoniae* strain AP76 seems to spread regularly to different body tissues of the pig during the acute phase of infection. The detection of the pathogen is not only limited to lungs and pleural cavity suggesting that it has invasive capacities. The pathogens ability to colonize the lungs and the degree of spleen colonisation are highly significantly correlated to the extent of spreading within the pig. In most cases *A. pleuropneumoniae* does not cause pathomorphological alterations in other organs than lungs and pleural cavity. Nevertheless, the strain used within this study seems to harbour the potential of causing lesion within these other organs as in five of eight animals inflammatory lesions were detected by histological examination. Further investigations are needed to examine underlying mechanisms of invasion involved in triggering disease of tissues other than the lungs, as well as, to identify the route of spreading, the ability of other strains and serovars for multi-organ spreading and the survival time of the agent when harboured in other tissues.

## Abbreviations

A.: *Actinobacillus*
Ab: antibody
cfu: colony-forming unit
cm^2^: square centimetre
Co.: company
EDTA: ethylenediaminetetraacetate
IHC: immunohistochemistry
kg: kilogram
m^2^: square meters
mg: milligram
mL: millilitre
NaCl: natrium chloride
PCR: polymerase chain reaction
pi: post-infection
SD: standard deviation
µm: micrometre
